# Pharmacokinetics and Bioavailability of Carprofen in Rainbow Trout (*Oncorhynchus mykiss*) Broodstock

**DOI:** 10.3390/pharmaceutics13070990

**Published:** 2021-06-30

**Authors:** Kamil Uney, Duygu Durna Corum, Ertugrul Terzi, Orhan Corum

**Affiliations:** 1 Department of Pharmacology and Toxicology, Faculty of Veterinary Medicine, University of Selcuk, Konya 42130, Turkey; 2 Department of Pharmacology and Toxicology, Faculty of Veterinary Medicine, University of Kastamonu, Kastamonu 37150, Turkey; ddurna@kastamonu.edu.tr (D.D.C.); orhancorum@kastamonu.edu.tr (O.C.); 3 Faculty of Fisheries, University of Kastamonu, Kastamonu 37150, Turkey; ertugrulterzi@gmail.com

**Keywords:** bioavailability, broodstock, carprofen, pharmacokinetics, rainbow trout

## Abstract

The aim of this study was to determine the pharmacokinetics of carprofen following intravenous (IV), intramuscular (IM) and oral routes to rainbow trout (*Oncorhynchus mykiss*) broodstock at temperatures of 10 ± 1.5 °C. In this study, thirty-six healthy rainbow trout broodstock (body weight, 1.45 ± 0.30 kg) were used. The plasma concentrations of carprofen were determined using high-performance liquid chromatography and pharmacokinetic parameters were calculated using non-compartmental analysis. Carprofen was measured up to 192 h for IV route and 240 h for IM, and oral routes in plasma. The elimination half-life (t_1/2λz_) was 30.66, 46.11, and 41.08 h for IV, IM and oral routes, respectively. Carprofen for the IV route showed the total clearance of 0.02 L/h/kg and volume of distribution at steady state of 0.60 L/kg. For IM and oral routes, the peak plasma concentration (C_max_) was 3.96 and 2.52 μg/mL with the time to reach C_max_ of 2 and 4 h, respectively. The bioavailability was 121.89% for IM route and 78.66% for oral route. The favorable pharmacokinetic properties such as the good bioavailability and long t_1/2λz_ for IM and oral route of carprofen suggest the possibility of its effective use for the treatment of various conditions in broodstock.

## 1. Introduction

Analgesic drugs are indispensable for pain management in mammals; however, their use in fish remains limited [1]. In recent years, with the increasing awareness of animal welfare, the management of pain and inflammation in fish under human care has been emphasized [2]. Although it is believed that there is no pain perception in fish, studies have shown that they have nociceptors responsible for transmission of painful stimulation, similar to mammals [3]. In various fish species, adverse changes in behavior and physiology in response to negative noxious stimuli were attenuated by the administration of analgesics [3]. Analgesic drugs are recommended for surgical operations, trauma-related injuries, cutaneous ulceration, and inflammatory lesions in fish [4]. Opioids and non-steroidal anti-inflammatory drugs (NSAIDs) are frequently used for pain control in fish [1]. In clinical trials, opioid use has caused side effects on cardiovascular and respiratory systems in fish; however, no adverse effects have been reported for NSAIDs [1]. NSAIDs have reduced postsurgical muscle damage in koi carp and minimal anesthetic concentration in goldfish and resulted in faster post-pain feeding in rainbow trout [3].

Pain and inflammation are associated with cyclooxygenase enzymes (COX-1 and COX-2), mostly COX-2, which is involved in the synthesis of prostaglandins (PGs) found at high levels at inflamed sites [5]. COX-1 is constitutively expressed in most tissues and synthesizes PGs that play a role in maintaining of normal physiological functions. COX-2 exists in an inducible form and its release increases during the inflammatory process in different cell types [6]. It has been demonstrated that the COX-2 enzyme, which has been identified in many fish species, plays a role in physiological and pathological processes [7,8]. The COX-1 and COX-2 enzymes found in rainbow trout are 77% and 83–84% homologous to those in mammals, respectively [9]. COX inhibitors, especially COX-2 inhibitors, reduce increased levels of PGs following the administration of lipopolysaccharides to different fish immune cells [8].

Carprofen is an arylpropionic acid class NSAID that prevents the synthesis of PGs by inhibiting the COX enzyme and has analgesic, antipyretic and anti-inflammatory properties [10,11]. NSAIDs with the COX-1/COX-2 selectivity ratio (the ratio of the NSAID concentrations required for inhibition of COX-1 and COX-2) of >1 are considered more potent in inhibiting COX-2 [12]. Carprofen in dog (1.75), cat (25.6) and sheep (5.3–6.3) has preferentially inhibition on COX-2 with the COX-1/COX-2 ratio of >1 [13,14,15]. Carprofen contains a single asymmetrical carbon atom and therefore exists in two isomeric forms, the S(+) and R(–) enantiomers, and commercially available products of carprofen are racemic (50:50) mixture of the two enantiomers. Carprofen is recommended for use in cattle, horse, dog and cat in musculoskeletal pain, surgery or trauma pain, mastitis, respiratory disease and osteoarthritis [11]. Carprofen showed an analgesic effect in the rainbow trout by shortening the feeding time period in the painful condition induced by acetic acid [16].

There are commercial formulations of carprofen in injectable and oral, and the use of carprofen in cattle (IV, SC), horse (IV), dog and cat (IV, SC, IM, oral) is recommended [17,18]. However, there is no drug formulation for the use of carprofen in fish. The development of new drug formulations should be based on the integration of pharmacokinetic, pharmacodynamic and clinical efficacy/safety studies [19]. Pharmacokinetics provides a mathematical basis to assess the time course of drugs and their effects in the body. It enables the absorption, distribution, metabolism, and excretion (ADME) processes to be quantified. A principal understanding of these parameters is required to design an appropriate drug dosage regimen for target species [20,21]. Although there are clinical studies of carprofen in fish, there are no pharmacokinetic studies. Therefore, pharmacokinetic and bioavailability studies are required to establish drug distribution in the body, calculate the effective and safe dosage, and determine alternative routes of administration [22].

Although the therapeutic effects of NSAIDs in fish are mostly investigated [1], pharmacokinetic studies are limited. Pharmacokinetic studies of ketoprofen in rainbow trout, ketoprofen, and meloxicam in Nile tilapia have reported short elimination half-life [2,23]. Studies in mammals and avian species have determined that the half-life of carprofen is much longer than those of meloxicam and ketoprofen [24,25,26,27]. Superior pharmacological properties of carprofen such as long elimination half-life, high bioavailability, and low risk of gastric irritation (ulcerogenic dose/anti-inflammatory dose ratio of 32) are advantageous (administration at wide dose intervals, less invasive procedures, and less stress and labor) for use in broodstock [10,28]. We hypothesized that carprofen would be used as a potential NSAID in rainbow trout broodstock by determining its pharmacokinetics and bioavailability for IM and oral routes. The aim of this study was to determine the pharmacokinetics and bioavailability of carprofen following intravenous (IV), intramuscular (IM) and oral routes to rainbow trout broodstock at temperatures of 10 ± 1.5 °C.

## 2. Materials and Methods

### 2.1. Chemical

The analytical standard of carprofen (>97%) was obtained from Sigma-Aldrich (St. Louis, MO, USA). Methanol (VWR International, Fontenay-sous-Bois, France) was used at analytical grade for high-pressure liquid chromatography (HPLC). Sodium acetate, n-butyl acetate, perchloric acid and acetic acid were purchased from Merck (Darmstadt, Germany). For the IV and IM injection, parenteral formulations of carprofen (50 mg/mL, Rimadyl, Injection Solution, Zoetis) was used. For the oral administration, tablet formulation of carprofen (50 mg, Rimadyl, Tablet, Zoetis) was used. The commercial formulations were diluted to a concentration of 20 mg/mL using sterile water for drug administrations.

### 2.2. Animals

In total, thirty-six clinically healthy rainbow trout (*Oncorhynchus mykiss*) broodstock with an average body weight of 1.45 ± 0.30 kg were used. Fish were determined to be free of bacteria and external parasites [29] and visually inspected for signs of illness, trauma, or poor body condition before inclusion in the study. The fish were procured from a local farm (Kastamonu, Turkey) and held in six ponds (500 L) in the farm, and each pond contained six fish. The fish were maintained in the ponds for 2 weeks for acclimatization. The fish were housed under normal daily lighting conditions and kept in the flow-through spring water (pH: 8.1 ± 0.2, water temperature: 10 ± 1.5 °C). Fish were fed with drug-free commercial fish feed (Sibal, Sinop, Turkey) every day. Fish were fasted for 12 h before and after drug administration. The study protocol was approved by the Kastamonu University Animal Experiments Local Ethics Committee, Turkey.

### 2.3. Drug Administration and Sampling

The rainbow trout broodstock were randomly divided into three groups equally according to the IV, IM and oral administrations of carprofen. The rainbow trouts received carprofen via IV (*n* = 12, caudal tail vein), IM (*n* = 12, epaxial muscle), and oral (*n* = 12, via the gastric gavage) administration at a dose of 2.5 mg/kg. The drug administration and blood samples were taken under MS-222 (tricaine methanesulphonate, 200 mg/L) anesthesia. Blood samples (0.6–0.9 mL) were collected into heparin tubes from the caudal vein of each animal by use of 22-G needles attached to 2 mL syringes. Blood samples were collected before carprofen administration (0 h) from all rainbow trout and following the IV, IM and oral administrations, at 0.25, 1, 4, 12, 48, 96, and 192 h from six trout and at 0.5, 2, 8, 24, 72, 144, and 240 h from another six trout. Therefore, a maximum of eight blood samples were collected from each trout for determination of carprofen concentrations. The collected blood samples were centrifuged at 4000× *g* for 10 min to obtain plasma and then stored at −80 °C until analysis.

### 2.4. Analytical Procedure

The plasma carprofen concentrations were determined using the HPLC-UV (Shimadzu, Tokyo, Japan) according to the previous method [24,30]. Briefly, 100 μL of trout plasma samples were transferred to 2 mL microcentrifuge tubes. Then, 150 μL acetate buffer (1 M, pH: 2.8) was added to 100 μL plasma and vortexed for 45 s, and 2 mL of n-butyl acetate was added. The tubes were mixed by vortexing for 45 s and centrifuged at 12,000× *g* for 15 min. The supernatant was transferred to auto-sampler vials, and 20 μL of this solution was injected into the HPLC system. HPLC system consists of a pump (LC-20AT controlled by the CBM-20A), an auto-sampler (SIL 20A), a column oven (CTO-10A), and a degasser (DGU-20A). The detection of carprofen was performed at 254 nm with an UV-VIS detector (SPD-20A). A Gemini^TM^ C18 column (250 × 4.6 mm; internal diameter, 5 μm; Phenomenex, Torrance, CA, USA), which was kept at 30 °C, was used for chromatographic separation. The mobile phase with a flow rate of 1 mL/min consisted of methanol (70%) and aqueous solution (30%, containing 50 μL of 0.2% perchloric acid in water).

Validation was performed according to EMA [31] guidelines for the chromatographic method. The stock solution of carprofen was prepared in methanol to obtain a concentration of 1 mg/mL. The stock solution was diluted to prepare working standards (0–20 μg/mL) and the calibration standards and quality control samples were prepared. The calibration standards (0.02–20 μg/mL) were prepared by the addition of different concentrations of working standard solutions containing carprofen into the blank trout plasma samples. Correlation coefficients for working standard dilutions and calibration standards of carprofen were determined as >0.9997 and >0.9993, respectively. The lower limit of quantification was 0.02 µg/mL for carprofen in trout plasma with the coefficient of variation less than 20% and the bias of ±15%. The quality control samples of carprofen at low (0.1 μg/mL), medium (1 μg/mL), and high (10 μg/mL) concentrations were used to determine the recovery, precision, and accuracy. The recovery of carprofen ranged from 92% to 102%. The intra-day and inter-day coefficients of variation and the bias of the assay of carprofen were ≤4.4%, ≤6.2%, and ±8.1%, respectively.

### 2.5. Pharmacokinetic Analysis

The plasma concentration–time curves of carprofen were plotted using WinNonlin 6.1.0.173 software (Pharsight Corporation, Scientific Consulting Inc., Gaithersburg, MD, USA). Plasma concentrations were presented as mean ± standard deviation (SD) values. Pharmacokinetic parameters of carprofen were calculated by the non-compartmental analysis using mean plasma concentration obtained at each sampling time after IV, IM and oral administrations [32,33]. The terminal elimination half-life (t_1/2λz_), area under the plasma concentration–time curve (AUC), AUC extrapolated from t_last_ to ∞ in % of the total AUC (AUC_extrap_ %), mean residence time (MRT), mean absorption time (MAT = MRT_IM,_oral − MRT_IV_), total clearance (Cl_T_ = Dose/AUC), volume of distribution at steady state (V_dss_= Cl_T_ × MRT) and bioavailability (F = AUC_IM,oral_ × 100/AUC_IV_) were determined. The AUC_IV_ and AUC_IM,oral_ were determined using the linear/log trapezoidal method and the linear up/log down method, respectively. The peak plasma concentration (C_max_) and time to reach C_max_ (T_max_) were determined via direct inspection on the plasma concentration–time curves.

## 3. Results

**Figure 1:**
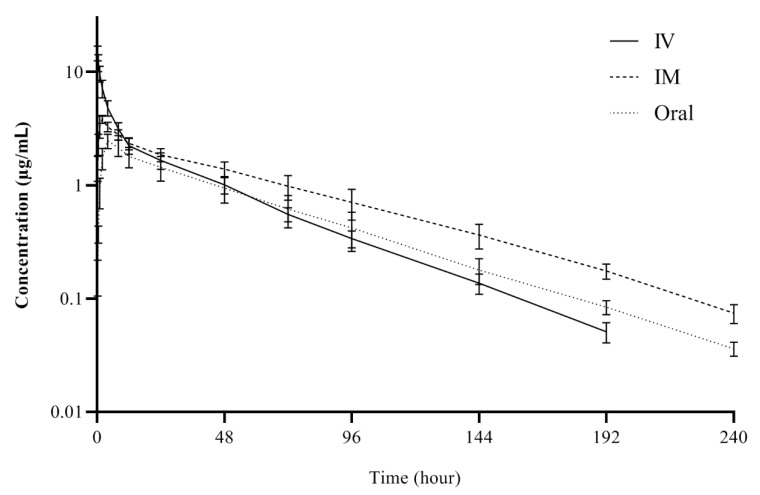
Semi-logarithmic plasma concentration-time curves of carprofen following intravenous (IV), intramuscular (IM) and oral administrations at a single dose of 2.5 mg/kg in rainbow trout (*Oncorhynchus mykiss*) broodstock at 10 ± 1.5 °C (mean ± SD, *n* = 6).

The semi-logarithmic plasma concentration–time curves and the pharmacokinetic parameters of carprofen following IV, IM, and oral administrations at a dose of 2.5 mg/kg in rainbow trout broodstock are presented in Figure 1 and Table 1, respectively. Following the IV, IM, and oral administration, at the first observational time point (0.25 h), the carprofen concentration was 14.71 ± 2.16, 1.44 ± 0.36, and 0.16 ± 0.06 μg/mL, respectively. Carprofen was measured up to 192 h for IV route and 240 h for IM and oral routes in plasma. The t_1/2λz_ was 30.66, 46.11, and 41.08 h for IV, IM and oral routes, respectively. Compared to IV administration, t_1/2λz_ after IM and oral administration prolonged by 50.4% and 34%, respectively. Carprofen for the IV route showed the Cl_T_ of 0.02 L/h/kg and V_dss_ of 0.60 L/kg. For IM and oral routes, the C_max_ was 3.96 ± 0.38 and 2.52 ± 0.26 μg/mL with the T_max_ of 2 and 4 h, respectively. The bioavailability was 121.89% for IM route and 78.66% for oral route.

## 4. Discussion

No adverse effects have been observed in rainbow trout broodstock after intravenous (IV), intramuscular (IM), and oral administration of carprofen at a dose of 2.5 mg/kg. No side effects have been reported in clinical studies with other NSAIDs such as ketoprofen and meloxicam in different fish species [1,2]. The use of carprofen is recommended at a dose range of 1–5 mg/kg in fish [1,3]. Following acetic acid-induced pain in rainbow trout, administration of 2.5 and 5 mg/kg of carprofen showed an analgesic effect. However, because the decrease in the activities of fish at a dose of 5 mg/kg has been reported [16], in this study, the dose of 2.5 mg/kg was selected. In addition, unlike other pharmacokinetic fish studies [34,35,36], repeated blood samples in this study were obtained. A similar design was used in pharmacokinetic studies of meloxicam and ketoprofen in fish [2,23]. Repeated handling of animals was quickly done without trauma by experienced personnel. Blood collections were performed rapidly under anesthesia (MS-222) to reduce possible stress response. The blood volume in fish is 3–7% of the total body weight, and the amount of blood taken at the sampling times should not exceed 15% of the total blood volume [37]. Fish were used for sampling 8 times at most and the total amount of blood (<7.2 mL) collected did not exceed 15% of the total blood volume.

The t_1/2λz_ of carprofen following IV administration in rainbow trout broodstock was 30.66 h at 10 ± 1.5 °C, which was similar to the previously reported in cow (30.7 h) [38], and sheep (26.1–33.7 h) [25], and longer than that reported in dog (8–11.7 h) [39,40], horse (21.9 h) [28] and cat (15.5 h) [41]. Fish are heterothermic animals and ambient water temperature significantly affects their physiology and metabolism, thus the pharmacokinetics of drugs [3,42]. The t_1/2λz_ of drugs in fish is prolonged with decreasing ambient water temperature [43]. It was surprising that t_1/2λz_ of carprofen was not longer in rainbow trout kept at low water temperature (10 ± 1.5 °C) than in mammals species such as sheep and cow [25,38]. Because the t_1/2λz_ is a hybrid parameter depending Cl_T_ and V_d_ [44], the different t_1/2λz_ of carprofen among species reflects the difference in Cl_T_ and V_d_.

The V_dss_ of carprofen in rainbow trout was 0.60 L/kg, which was larger than that previously reported in horse, cow, sheep, and dog (0.09–0.23 L/kg) [25,28,38,40]. The binding of drugs to plasma proteins and body composition affect the distribution volume [45]. Because NSAIDs are highly bound to plasma proteins, their V_dss_ is generally low [46]. The binding ratio of carprofen to plasma proteins is not known in fish, but it is highly protein-bound (>99%) in mammals [10]. The amount of total plasma protein in trout is half compared with that in mammals; therefore, the binding ratio of drugs to plasma proteins in fish is lower than in mammals [37]. Acidic drugs such as naproxen have a lower binding ratio to plasma proteins in trout (94.6%) than in mammals (>99.9%) [47]. Because carprofen is also weakly acidic (pKa: 4.3) [40], its binding to plasma proteins may be low in trout. The larger V_dss_ in rainbow trout may be owing to the variation in body composition (anatomical and physiological) and in the ratio of binding to plasma proteins between species. The Cl_T_ following IV administration of carprofen in trout was 0.02 L/h/kg, which was similar to that previously reported in dogs (0.017 L/h/kg) [40], and was greater than that reported in cows, sheep and cats (0.002–0.005 L/h/kg) [25,38,41]. In fish, the excretion of drugs can occur through the gills, kidney and bile [48,49]. Carprofen is excreted via urine (8–70%) and bile (35–75%) mostly as conjugate metabolites after undergoing varying rates of phase I and phase II reactions in mammals [10,50]. In addition, the ratios and pharmacokinetics of S (+) and R (−) enantiomers in the body after racemic carprofen administration differed among species [41]. Although the metabolism of carprofen in fish is unknown, it has been stated that there are phase I and phase II biotransformation enzymes in fish [37]. Variability of Cl_T_ among species may be due to the difference in enantioselectivity, metabolism, and excretion pathways of carprofen.

Compared to IV administration, t_1/2λz_ after IM and oral administration in rainbow trout was prolonged by 50.4% and 34%, respectively. The t_1/2λz_ of carprofen was found to be similar after oral (6.7–9.3 h) and IV (8 h) administration in dogs and longer after IM (29.4) administration than after IV (21.9) administration in horses [28,39]. The longer t_1/2λz_ after extravascular administration than after IV administration may result from the flip–flop phenomenon, precipitation of the drug or physiological changes in fish due to low water temperature. The flip–flop phenomenon, in which the rate of absorption of a drug is slower than the rate of elimination, often occurs with extravascular administration. In this case, the MAT value is expected to be longer than the MRT value after IV administration [51]. However, the longer MAT values than MRT_IV_ obtained in this study do not support the flip–flop phenomenon. It has been stated that the precipitation or crystallization of carprofen at the IM injection site in horses can be a depot for continuous absorption [28]. In fish, low water temperature causes slowing of heart rate, tissue perfusion, and gastrointestinal tract movements [52,53]. The long t_1/2λz_ after the extravascular administration may be due to the fact that the fish were kept at low water temperature (10 ± 1.5°C).

The C_max_ (3.96 μg/mL) of IM route at 2.5 mg/kg dose in rainbow trout broodstock was lower than that previously reported in horse (2.2 μg/mL, 0.7 mg/kg) [28], and quails (20.67 μg/mL, 10 mg/kg) [24]. The T_max_ of IM route in trout was 2 h, which was longer than reported in quails (0.29 h) [24] and shorter than reported in horse (10.6 h) [28]. The C_max_ (2.52 μg/mL) of oral route at 2.5 mg/kg dose in rainbow trout was lower than that previously reported dogs (5.43–35.30 μg/mL, 0.7–4 mg/kg) [39] and higher than that reported in quails (6.53 μg/mL, 10 mg/kg) [24]. The T_max_ of oral route in trout was 4 h, which was longer than reported in quails (0.92 h) [24]. In dogs, the T_max_ of oral route ranged from 1.25 to 3.75 h [39]. The bioavailability of carprofen after IM administration in rainbow trout was 121.89%, which was higher than that in horse (40–70%) [28]. In trout, the bioavailability of carprofen after oral administration was good with 78.66%. Carprofen is well absorbed from the gastrointestinal tract, and its oral bioavailability in dogs and horses is from 75 to 112% [10,40]. The bioavailability following extravascular administration is generally ≤100% [44]. The bioavailability of carprofen after IM administration in trout was 121.89%, which was close to 100%. The bioavailability higher than 100% may be due to reasons including experimental errors (e.g., analytical artefacts, vehicle effects) and common mechanistic explanations (e.g., different groups of animals for IV and extravascular route of administration) [44]. In this study, when experimental errors are ruled out with controlled study, different groups of animals for IV and IM route of administration might have result in the observed bioavailability higher than 100%.

The t_1/2λz_ of carprofen in different administration groups ranged from 30.66 to 46.11 h, which was longer than that reported in Nile tilapia for meloxicam (1.36–1.80 h) [23] and in rainbow trout for ketoprofen (3.91–4.40 h) [2]. Minimal handling is required for medical treatment in fish to minimize the risk of stress, trauma, and infection [23]. The long t_1/2λz_, long-lasting effect and less labor and adverse effects following a single dose of carprofen favor its use in broodstock. However, the long t_1/2λz_ of the drug may increase the risk of drug accumulation and toxicity on repeated dosing. The temperature-dependent pharmacokinetics of drug in fish is an important consideration for drug residues. Therefore, for farmed fish, the withdrawal times based on temperature-dependent residue levels are determined in degree-days (°C × days) and set a withdrawal period not less than the minimum of 500 degree-days [42,54]. Because carprofen shows long t_1/2λz_ in fish, repeated dosing may result in residual risk and adverse effects. After prolonged exposure (4–14 days) to NSAIDs (diclofenac, salicylate, ibuprofen, mefenamic acid) following bath administration, adverse effects on reproduction, osmoregulation, homeostatic functions, and immunity have been observed in fish [55,56,57,58]. There is insufficient data regarding the safety of carprofen in fish. Thus, the safety of carprofen after single and repeated administrations should be established before the administration in fish.

In fish, there have been several drug administration routes such as injection, oral, and bath [59]. Although the administration of drugs via injection is very effective, it is not practical due to the need for more time and labor such as catching, handling and injecting large number of fish, and therefore it is generally preferred in broodstock and ornamental fishes [59,60]. In this study, because the IM administration of carprofen exhibited good bioavailability and long t_1/2λz_, the commercial formulation of carprofen administered to fish might be preferred for use in broodstock. The oral administration via gastric gavage was used to determine the exact dose and to prevent possible drug loss due to feed. However, the medicated feed is preferred due to the ease of oral administration to fish. In this study, favorable pharmacokinetic properties (good bioavailability and long t_1/2ʎz_) of carprofen following oral administration can provide fundamental data for the development of new formulations in the form of medicated feed in fish. In addition, because the bath administration is the most preferred method for fish due to its ease of application [59,60], there is a need for new drug formulations of carprofen in the form of bath.

## 5. Conclusions

The favorable pharmacokinetic properties such as the good bioavailability and long t_1/2λz_ for IM and oral route of carprofen suggest the possibility of its effective use for the treatment of various conditions in rainbow trout broodstock. In addition, these properties may be advantageous such as the administration at wide dose intervals, less invasive procedures, and less stress and labor for use in rainbow trout broodstock. However, further studies are required to evaluate the clinical efficacy and safety of administering carprofen before use.

## Figures and Tables

**Table 1:** Plasma pharmacokinetic parameters of carprofen following intravenous (IV), intramuscular (IM) and oral administrations at a single dose of 2.5 mg/kg in rainbow trout (*Oncorhynchus mykiss*) broodstock at 10 ± 1.5 °C.

Parameters	IV	IM	Oral
t_1/2λz_ (h)	30.66	46.11	41.08
AUC_0-last_ (h * µg/mL)	157.24	189.47	123.32
AUC_0__–__∞_ (h * µg/mL)	159.49	194.40	125.46
AUC_extrap_ (%)	1.41	2.54	1.70
MRT_0__–__∞_ (h)	38.55	66.86	58.78
MAT (h)	-	28.31	20.23
Cl_T_ (L/h/kg)	0.02	-	-
V_dss_ (L/kg)	0.60	-	-
C_max_ (µg/mL)	14.71 ± 2.16 *	3.96 ± 0.38	2.52 ± 0.26
T_max_ (h)	-	2	4
F (%)	-	121.89	78.66

* Plasma concentration of carprofen at first sampling time following IV administration. t_1/2__λz_, terminal elimination half-life; AUC, area under the plasma concentration-time curve; AUCex_trap_ %, area under the plasma concentration-time curve extrapolated from tlast to ∞ in % of the total AUC; MRT, mean residence time; MAT, mean absorption time; Cl_T_, total clearance; V_dss_, volume of distribution at steady state; C_max_, peak plasma concentration; T_max_, time to reach C_max_; F, absolute bioavailability.

## Data Availability

The data presented in this study are available on request from the corresponding author.
